# Advanced Bioelectrical Signal Processing Methods: Past, Present, and Future Approach—Part III: Other Biosignals

**DOI:** 10.3390/s21186064

**Published:** 2021-09-10

**Authors:** Radek Martinek, Martina Ladrova, Michaela Sidikova, Rene Jaros, Khosrow Behbehani, Radana Kahankova, Aleksandra Kawala-Sterniuk

**Affiliations:** 1 Department of Cybernetics and Biomedical Engineering, VSB-Technical University Ostrava, FEECS, 708 00 Ostrava, Czech Republic; martina.ladrova@vsb.cz (M.L.); michaela.sidikova@vsb.cz (M.S.); rene.jaros@vsb.cz (R.J.); radana.kahankova@vsb.cz (R.K.); 2 College of Engineering, The University of Texas in Arlington, Arlington, TX 76019, USA; kb@uta.edu; 3 Faculty of Electrical Engineering, Opole University of Technology, Automatic Control and Informatics, 45-758 Opole, Poland

**Keywords:** biomedical signals, signal processing, electromyography, electroneurography, electrogastrography, electrooculography, electroretinography, electrohysterography

## Abstract

Analysis of biomedical signals is a very challenging task involving implementation of various advanced signal processing methods. This area is rapidly developing. This paper is a Part III paper, where the most popular and efficient digital signal processing methods are presented. This paper covers the following bioelectrical signals and their processing methods: electromyography (EMG), electroneurography (ENG), electrogastrography (EGG), electrooculography (EOG), electroretinography (ERG), and electrohysterography (EHG).

## 1. Introduction

This work is the last and third part of the review paper regarding the most recent and the most advanced processing methods for bioelectrical signals. Part I concerned heart signals [1]; Part II— brain signals. This part (Part III) is about analysis methods for other bioelectrical signals such as electromyography (EMG), electroneurography (ENG), electrogastrography (EGG), electrooculography (EOG), electroretinography (ERG), and electrohysterography (EHG).

This study mainly presents the so-called informatic-related advanced signal processing methods, which are used for digital, post-ADC signals. Purpose of these methods is to extract critical information regarding the health condition of the patients, which is contained in the signals.

Analysis of bioelectrical signals is a very challenging task, as they are all prone to various disturbances and artifacts occurrence. The use of sophisticated signal processing methods may improve these signals’ quality and may make them more suitable for various diagnostics’ purposes.

The methods described in this work are based on authors’ subjective decisions. It was impossible to mention all the methods, mostly due to the rapid development of this scientific area.

## 2. Electromyography

Electromyography (EMG) is a diagnostic method, which enables recording of bioelectric signals resulting from the activities of the skeletal muscles [2,3]. It is often performed while stimulating the relevant motor and peripheral nerves. The measurement may be carried out either in an invasive or surface-based way, at the level of a single muscle fiber, single motor unit, or the entire muscle. The processing of information from the EMG enables diagnostics of muscle and neuromuscular disorders, or to analyze or use the EMG for the rehabilitation or limb prostheses control purposes [2,4,5].

**Figure 1:**
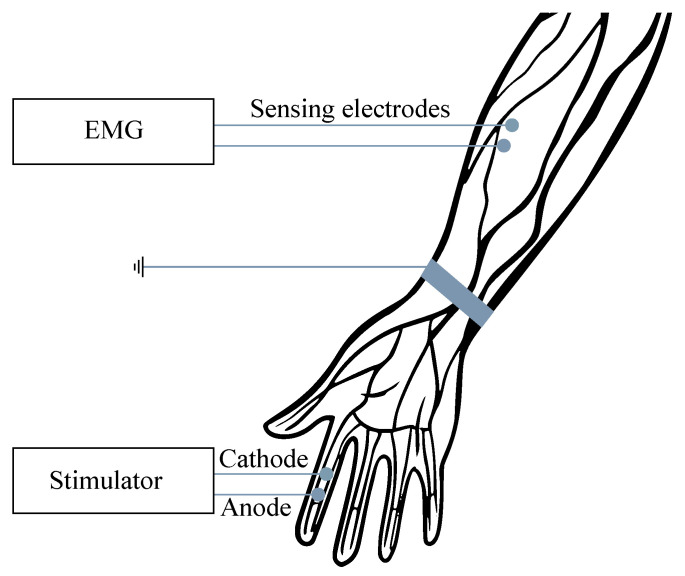
Example of the surface EMG signal measurement.

For the examination purposes, the monopolar or bipolar electrodes may be used and, in some cases, the combination of intramuscular and surface electrodes can also be involved. For recording from the surface, the so-called multi-electrodes are used, when the electrodes are placed in slots on the silicone mat, either in a row (strips) or matrix (grids). In order to reduce the signal interference, the power supply voltage with the right foot (same as with the ECG) can be applied, when the grounding electrode is placed sufficiently far from the scene of the recording (see Figure 1). The EMG frequency ranges vary from 0.01 Hz to 10 kHz, depending on the type of examination (invasive or noninvasive). The most useful and important frequency ranges are within the range from 50 to 150 Hz [6,7,8,9,10].

It is also important to mention modern solutions in healthcare, which involve measurement of among the others EMG signals—wearable and wireless body-area networks (BAN or WBAN), which integrate multiple sensors for motion, inertial, and biosignals with low-power radios. They usually work in real-time. In order to make such systems efficient compressed sensing, or compressive sampling (CS), is applied. The CS is a method for data acquisition, where only few incoherent measurements are required in order to compress sparse in some domain signals [3,11,12,13]. In [11,12,13], the authors showed interesting solutions for compressing both ECG (electrocardiography) and EMG data. In [3], the authors focused on CS for the purpose of the EMG signals reconstruction in order to design efficient low-powers WBANs. Their work compares four different algorithms applied in practical implementations.

### 2.1. EMG Recordings

The EMG recording methods can be divided into the two main categories—motor unit action potential (MUAP) and compound muscle action potential (CMAP) [14].

#### 2.1.1. MUAP

**Figure 2:**
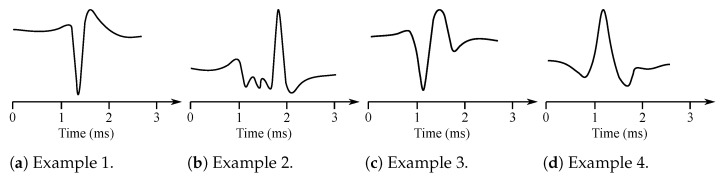
Examples of the MUAP potentials.

Muscles consist of motor units, which are the smallest possible portions of muscles, which can be activated [15]. The sum of the action potentials of the respective muscle fiber can be measured invasively with needle electrodes placed directly into the muscle in the area of interest. The signal is thus obtained with the superposition of the individual MUAP [14]. Therefore, it is necessary to further disassemble this signal on the contributions of each motor unit. Typical MUAP are two-phase or three-phase, lasting approximately 3–15 ms and reaching an amplitude between 100 and 300 µV (see Figure 2).

They repeat 6–30 MUAP per second. The shape of the MUAP depends on the type of the used needle electrode, its location in relation with the motor unit, and the resolution of the electrical activity of the electrodes. Furthermore, the wave shape is different in case of some particular diseases such as, e.g., neuropathy or myopathy. The neuropathy causes slow conduction or unsynchronized activation of muscle fibers within a single motor unit. The MUAP has in this case a greater amplitude. The myopathy is manifested with loss of muscle fibers when neurons are non-functional. Then, the fragmentation of the MUAP occurs due to asynchronous activation, which leads to the multi-phase MUAP [6,7,16].

#### 2.1.2. CMAP

**Figure 3:**
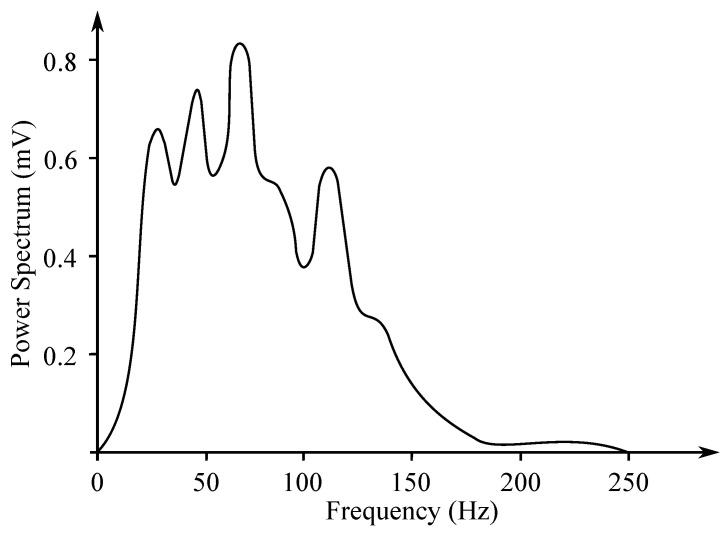
Example of the surface EMG frequency spectrum (based on the work in [18]).

Another method of EMG measurement is the recording of the entire muscle activity, which consists of the sum of the individual MUAP. It is measured with the surface electrodes and its analysis is far more complex, as it uses a frequency range from 10 Hz to 1 kHz (see Figure 3). The sampling frequency of 2 kHz is usually applied. The signal reaches up to 10 mV amplitude. Due to the possible muscle disease, the amplitude of the signal is visibly reduced. If the amplitude gradually decreases, it causes a problem with the transmission at the neuromuscular junction. In case of the demyelination of nerves, contraction of the muscle fibers delays with a normal amplitude of the responses [17].

When measuring the EMG, the so-called maximum free contraction of the muscle is evaluated. The required strength of contraction is achieved with the gradual activation of motor units. The number of activated units required for this contraction varies depending on the size of the muscles. First, there are always activated motor units innervating a smaller number of muscle fibers, increasing the need for contraction, will then engage the drive innervating a larger number of threads. This allows slowing the escalation of contraction. The muscles are never able to achieve a constant force contraction, due to the certain fluctuations in action potential propagation time of the motor unit and the diversity of reactions of the muscle fibers to tripping up. Oscillations were detected on the frequency of contraction of 1–2 Hz [6,7,16].

### 2.2. Clinical Applications

The EMG method is an important tool for assessment of neuromuscular disorders, even in a clinically normal muscle. It is a low-risk procedure associated with rare complications, such as bleeding, infection, or nerve injury [19,20,21]. In clinical practice, it is possible to interpret both the appearance of EMG signal waveform and the sound generated by an audio amplifier. During the EMG measurement, a healthy muscle tissue at the resting state should be (electrically) silent, with the exception of the area of the neuromuscular junction [2,4]. The response related to incomplete relaxation (due to patient’s inability to stay relaxed) differs from pathological spontaneous activity by its rhythmicity. The approach used in examining the EMG varies depending on the disorder type, patient’s age, and ability to cooperate. Based on that, there are several variables such as number of electrodes, their locations and placement, or their type. When interpreting the EMG results, a variety of factors must be taken into account, including patient’s health history, current health condition, results of other relevant diagnostic methods—such as nerve conduction studies, imaging methods (e.g., magnetic resonance imaging or ultrasound), muscle and nerve biopsy, etc. [2].

The EMG method can be incorporated in a variety of applications. For example, as a diagnostic method or a research tool for fields related with kinesiology, motor control-related, or neuromuscular disorders. This method is also useful in prosthetic device control, especially prosthetic arms or lower limbs [19]. Nevertheless, the EMG can only be applied in order to assess primary myopathic conditions related to electrical activity of the muscles. Therefore, for the appropriate evaluation of most neuromuscular disorders, it is necessary to also measure the electrical activity of the nerves that generate electrical signals to control the muscle response. Therefore, the EMG is usually performed simultaneously with the so-called nerve conduction studies (NCSs), which enable measurement of the nerves conductivity. The needle-based EMG and the NCSs are tested in case the patient suffers from neuromuscular disorder symptoms, such as limb pain or weakness, muscle paralysis, or spasms [21,22,23]. Moreover, the examination is also beneficial in diagnosing the nerve compression or injury (e.g., carpal tunnel syndrome or sciatica), and other neuromuscular disorders, such as amyotrophic lateral sclerosis (ALS), myasthenia gravis, and muscular dystrophy [22].

The EMG is a low-risk procedure, and complications during its performance are very rare. There is, however, a small risk of bleeding, infection, and nerve injury in a location where a needle electrode is inserted. When muscles along the chest wall are examined with the needle electrode, there is a very small chance that it could cause air to leak into the area between the lungs and chest wall, causing a pneumothorax (lung to collapse) [19,20,21].

### 2.3. EMG Signal Processing

The EMG signal can be considered to be challenging from the perspective of the large range of interference removal (as summarized in Table 1) due to its wide frequency range. The significant interferences of the surface EMG signal are the motion artifacts, which are very difficult to remove and occur in almost all biomedical signals. They are caused by the sensor movements in the area of interest which increase due to the direct press onto the sensor, fast movements of the body parts where the sensor is fixed or due to the changes in the balance of the electrode–skin interface caused by the muscle contraction resulting in volume changes. These artifacts mean the issue occurring during measurement of dynamic activities. The motion artifacts typically have the high-amplitude peak beside the EMG signal but they occur at the low frequencies, so it is possible to remove them using the high-pass filter which does not affect the measured frequency range of the EMG signal [24,25].

Another type of artifact present in the EMG data is the signal contamination with the signal of the nearby muscle, which is not in the area of interest, these are the so-called “cross-talk” signals. The data is then distorted in both amplitude and time duration of the EMG signal. This artifact can be reduced using reference electrodes which enable comparison of the amplitudes of the generated myopotentials and evaluation of the signal course in the area of interest. In case of the sensor disconnection or the signal amplitude overdoing, the signal saturation can originate which results in the distortion of the high amplitudes. Therefore, it is important to control the contact of the electrode and skin surface, reduce the gain of the amplifier or transfer the electrode to another place on the tested muscle where the amplitude can be reduced.

As in the case of the EEG recordings, the EMG signal is also significantly influenced with the ECG signal, which is at its most visible during the measurement of the muscles of the upper parts of the body. The problems are the high amplitude of the ECG signal against the EMG signal and the overlapping of their frequency spectra, so it is not possible to use the ordinary filtering methods in order to remove these artifacts. One of the ways is to locate the EMG electrodes as far from the heart as possible [26].

From the technical artifacts, the EMG signal can be corrupted with the PLI, electromagnetic interference of the other present sources, and components of the recording system. For the high-frequency noise suppression, the low-pass filters with the cut-off frequency higher than the EMG frequency range can be successfully applied [27,28,29].

### 2.4. EMG Processing Methods

In case of the EMG signal noise reduction, the classic frequency-based methods are not very suitable because the signal is non-stationary. However, these methods are often used for their easy implementation. A problem begins in particular during the elimination of noise signals which overlap with the desired EMG signal in the frequency band [24,26]. Table 1 summarizes the most common processing methods of the EMG data.

It is also important to mention, beside the methods described below, some interesting studies, where the EMG data has been analyzed. In Kawala-Janik et al. [5], a customized threshold-based method, previously tested on the EEG data (see in [30,31]) has been applied for the pattern recognition purposes. In [14], implementation of fractional filtering as an alternative to the traditional, integer-order filters in analysis of the EMG data was in detail presented. The obtained results were interesting and promising.

#### 2.4.1. Digital Filtering

The most common way for noise removal in the EMG signals is filtering. Application of various digital filters is quite a simple and fast method for the purpose of preprocessing step for the further analysis (e.g., muscular fatigue detection, limb movements, or emotions recognition). Usually, the BPF with the cut-off frequencies of 20 Hz and 400–600 Hz is used for the demarcation of the physiological frequency band of the EMG signal and removing the both high- and low-frequency interference. Then, the BSF with the cut-off frequencies 49–51 Hz is included in the preprocessing string in order to remove the PLI [8,10,32,33].

#### 2.4.2. Adaptive Noise Canceler

The Adaptive Noise Canceler (ANC)-based filtering can be successfully used for the purpose of the PLI or the ECG artifacts elimination. Soedirdjo et al. [34] used the ANC with the LMS adaptive algorithm and synthetic reference. The ANC might not be sufficient as the fundamental frequency of the PLI deviates up to ±1% from its nominal frequency, so a synthetic reference with varying frequency and phase is proposed. In comparison with the other methods tested by them (such as notch filter, Keshtkaran and Yang’s adaptive filtering, and spectral interpolation), the applied ANC resulted with the best SNR improvement. In case of the ECG artifact removal [35,36], the easiest way was to record the ECG signal and to use it as the reference input for the ANC.

#### 2.4.3. Wavelet Transform

The WT filtering method is frequently used in the EMG signal processing because of its non-stationary character and the ability of the method to distinguish data well in both frequency and time domain. Hussain et al. [37] used many wavelet functions in order to test the WT for the purpose of noise reduction in the surface EMG signals (Daubechies, symlet, Meyer). The wavelet Db2 seems to be the most powerful tool for the signal denoising using the WT. Jiang et al. [38] compared the different types of thresholding—Universal, SURE, Hybrid, and Minimax. Their experiments proved that the denoised EMG was insensitive to the selection of the denoising methods.

#### 2.4.4. Independent Component Analysis

Howard et al. [39] proposed a tool for reduction of the cross-talk signals from the EMG recordings via the ICA method. The EMG signal is suitable for using the ICA because it satisfies all its criteria (components must be statistically independent and the independent components must be non-Gaussian), where each muscle can be assumed to be an independent source, as the motor units in each muscle are well separated from the other muscles, and the finite sum of the MUAPs is non-Gaussian. Naik et al. [40] used a multi-run ICA method in order to eliminate the cross-talk signals obtained from the surface EMG and analyzed the number of sources of the signal. The multi-run ICA is the process where the ICA algorithm is computed many times—at each instance, so as a result—the different mixing matrices are obtained.

#### 2.4.5. Empirical Mode Decomposition

The EMD method was used for the purpose of decomposition of the EMG signal to the IMFs which were then processed with the implementation of other filtering methods and then, the denoised signal was reconstructed [41]. Andrade et al. [42] implemented the EMD in combination with the soft thresholding. The EMD was compared also with the WT using many types of maternal wavelets. Both of the proposed methods reduced the total power of the noise and at the same time preserved the majority of the energy of the signal.

Mishra et al. [43] proposed a simple technique with the application of the improved EMD (IEMD) in conjunction with four different features, which was used for the analysis of amyotrophic lateral sclerosis (ALS) and the normal EMG signals. The EMD method followed with the median filter has been employed for removal of the impulsive noise from the IMF components generated through the EMD. The filtered IMF components are summed together in order to generate a new signal. The EMD process is further applied to the new EMG signal to generate improved IMFs called the IEMD method. A new technique based on the IEMD algorithm was proposed for the first time, which enabled the choice of the window size of the applied median filter.

The EMD and the improved EMD, called the Ensemble EMD (EEMD), enable overcoming the limitation of the mode mixing routinely induced with the regular EMD, and is also used by Zhang et al. [44]. If the PLI component was present in the IMF, the notch filter was applied to the IMF. In order to reduce the white noise, a similar approach to the wavelet-based denoising methods can be implemented, including soft or hard thresholding. The BW components involved mainly the higher-order IMFs, which can be assessed by applying the LPF to the IMFs. Then, the signal can be reconstructed from the IMFs.

#### 2.4.6. Hybrid Methods

Abbaspour et al. [45] proposed a combination of the WT and the ICA methods for the purpose of the ECG artifacts elimination. The first step of the method was a wavelet decomposition (with the Db4 wavelet) in order to create 8 levels of the raw signal. After the wavelet coefficients were calculated, the ICA method was applied to the multidimensional data produced with the WT. Then, the independent components were classified automatically as either EMG signal or ECG artifact. The implementation of such filtering of the signals provided satisfactory results in the SNR improvement. The matrix of signals was obtained from five sensors—three of them were attached in ideal locations and two others were placed non-ideally in order to gather the cross-talk data. Through a combination of the channels, the three final signals were processed with the applied ICA method. The obtained results were identified as a successful distinction between individual muscle activation.

Ren et al. [46] removed the PLI using the above mentioned method in the contrary sequence. First, the ICA decomposition was done, when the independent components containing the PLI were filtered with the WT and the MUAP components were retained, which was the aim of the study. The white noise was then removed also using the WT. The hard thresholding was used for the WT and the signal was reconstructed with the inverse discrete WT.

## 3. Electroneurography

The electroneurography (ENG) is a method used to visualize directly recorded electrical activity of neurons in the central nervous system (CNS), which consists of the brain and the spinal cord or in the peripheral nervous system (PN) consisting of the nerves and nodes. The electroneurography is similar to the electromyography (EMG), but it is used in order to visualize the muscles activity as it is used to measure the conduction velocities and latencies in peripheral nerves by stimulating a nerve at different points along it [47].

The first ENG from a single nerve fiber was recorded by Edgar Adrian in 1928 using Lippmann’s electricity meter [48]. In 1953, the first Iridium recording micro-electrode was developed [49]. The first simultaneous recording with the implementation of the multiple units with a use of the multi-electrode set, which was performed on a patient during brain surgery, was published by Marg and Adams in 1967 [50].

The ENG is usually obtained through recording with the electrodes placed in the nervous tissue. The electrical activities generated with the neurons are recorded with the electrodes and are then transmitted to a collection system, which usually allows visualization of the activity of the neuron. Each vertical line in the electroneurogram represents only one neuron action potential. Depending on the accuracy of the electrode applied for the recording of the nerve activity, the obtained electroneurogram signal may contain the activity of one to thousands of neurons. The researchers adapted the accuracy of various electrodes either by focusing on the activity of the single neuron or on the general activity of a group of neurons, and both strategies have their advantages and disadvantages.

### 3.1. ENG Recording

**Figure 4:**
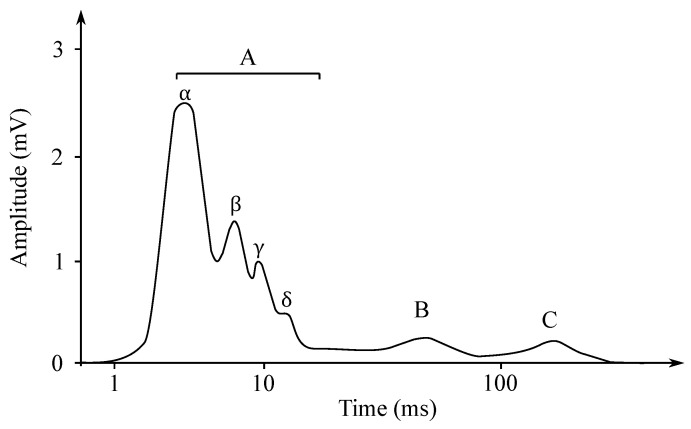
The ENG mixed nerve.

Individual nerve fibers conduct excitation at different paces. If the action potential of the whole nerve is read as the total action potential, then the obtained electroneurogram is a curve with several characteristic peaks. Each wave corresponds with the one type of the nerve fiber, such as, e.g., type A fibers, which are fibers mediating the movements of the skeletal muscles and they are led at the speed of approximately 70–120 m/s. In contrast, the type C fibers are fibers that mediate the feeling of heat or pain and are led at the speed of only 0.5–1 m/s [51]. In Figure 4, it is possible to see an ENG mixed nerve, where the category of the A fibers—myelinated, 4 subgroups, and the category the B fibers—myelinated preganglio vegetative and the category of the C fibers—unmyelinated postganglionic sympathetic fibers (Cs), centripetal pain fibers Cd.r—dorsal roots.

**Figure 5:**
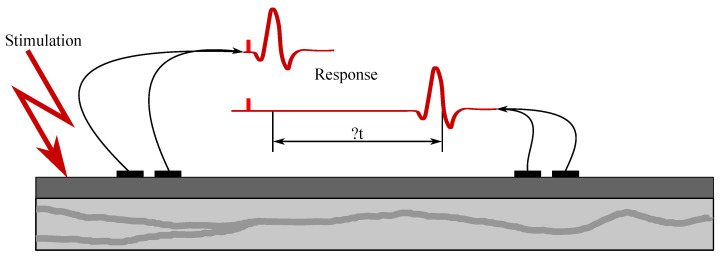
Sample ENG measurement.

Examination of their conductive function (excitation conduction velocity together with attenuation characteristics). In Figure 5, the ENG measurements were presented.

The resulting signal and the contribution of individual neurons (i.e., the amplitude and morphology of the ENG signal) are affected by the type and location of the neural fiber(s) and their proximity to the measuring electrode. The shape of the signal and the associated content is also influenced by the configuration of the device (e.g., bipolar or unipolar measurement, common reference or ground, etc.), as well as size and shape of the electrode’s active contact with the skin and its placement. Therefore, the recorded signal can consist of single peaks or it can be a composite of several action potentials. Its amplitude can thus range from 1 to 100 mV and its frequency from a few Hz to 10 kHz [47].

### 3.2. Clinical Applications

The ENG usually refers to the recordings obtained from the axon bundles in the peripheral nerves. In clinical practice, the ENG waveform can be displayed on a screen, converted to a sound and played through an audio amplifier, or used as a control signal for various neural prostheses or other devices. The ENG is recorded by means of electrode(s) placed in near proximity with the neurons of interest in order to cover their activity [52].

The ENG can be used for various purposes. For example, it is a valuable diagnostic tool for the assessment of various movement-related disorders [53] (e.g., ALS [54], multiple sclerosis (MS) [55], in biofeedback (e.g., in the closed loop systems applied for the end organ stimulation), for the assessment of muscles conditions, and for the purposes of control of the orthotics and prostheses [56,57,58].

Both ENG and EMG signals consist of various signals from several sources, including the desired ones from individual neurons and others, considered as noise, from surrounding tissue, organs, devices, or environment. The ENG signal can thus be considered as challenging in terms of signal processing and noise removal mostly due to its wide frequency range and the interference accompanying its acquisition. The main interference of the ENG signal sensed on the skin surface are the motion artifacts, which are difficult to remove. They are caused by the movements of the sensor, which are increased by pressing or otherwise manipulating with the sensor, rapid movements of the parts of the body where the sensor is mounted, or changes in the balance of the electrode–skin interface caused by the muscle contraction leading to the changes in volume. It is possible to remove them using inter alia high-pass filters, which do not affect the measured frequency range of both EMG and ENG signals [59,60,61].

In general, the raw ENG signal obtained must be first preprocessed before any further processing, extraction or analysis takes place. The preprocessing phase usually includes the signal amplification and basic band pass filtering defined according to the signal’s characteristics. This step is necessary to remove the unwanted signals, such as the EMG noise, nerve tissue background noise, motion artifacts, or conduction noise. The ENG device typically includes an input amplifier which enables the attenuation of the signal in the specific frequency bands, most frequently defined as high pass (0.01–1000 Hz), a low pass (500–10,000 Hz), and a notch (50 or 60 Hz. Moreover, the recording system is constructed in a way to provide a low noise (<2 mVpp), high normal mode rejection ratio (CMRR > 90 dB), high input impedance and high gain (1000–500,000) bandwidth differential recording capability (0.01–10 kHz). The preprocessed ENG signals are then digitized and stored or transmitted to the computing machine for further analysis and/or simply displayed on the screen [52,62].

### 3.3. ENG Processing Methods

The raw ENG signal contains very valuable information, although the extraction of this information from the recorded signal usually requires implementation of various online or offline signal processing or machine learning procedures [63]. Such signal processing methods usually include analysis in the time and frequency domains, or the mixed mode methods. For example, the frequency domain methods include the Laplace, Fourier, and Z-transforms, or calculations of power spectral density and signal phase. The time domain approaches may include the time-series analysis (e.g., moving averages or cross-correlations). The mixed mode methods are methods such as the band-pass filtering, waveform analysis, short Fourier transform, etc. The machine learning procedures, which have become very popular, are algorithms used for the detection of the statistical regularities in the specified data. They include learning information obtained from the previous data or generating (predicting) the new data. The most common methods used in the ENG signals processing include the principal component analysis (PCA), the independent component analysis (ICA), the auxiliary vector machines (SVM), and the artificial neural networks (ANN) [62,64,65].

Robert et al. [66] developed a new scheme for increasing the compression and interpretability of the multichannel bio-potentials obtained from the implanted nerve sensors. Spatial and temporal correlations between the samples were determined to find the appropriate patterns associated with the external stimulation affecting the scanned tissues. The applied time analysis included peak detection and sorting. The spatial processing was based on the ICA analysis of the observed nerve activity. Experiments using modeling and simulations were performed in order to test the ability of the system to assign the observed potentials to the applied external stimuli [65].

The ENG processing frequently uses a sequence of steps to remove noise and to find the temporal and spatial patterns of the processed signals. Both linear and nonlinear filtering techniques are used for the noise removal. Selection of the processing/extraction method depends on the feature one needs to obtain. For example, the peak frequency of the neural activity, which can be either instantaneous or calculated in a defined time window. To detect peak events in noisy signals, simple thresholding or the Schmitt trigger can be used. These methods require selecting a threshold value, commonly set as multiple value of the resting activity magnitude (e.g., three times the standard deviation of the signal). The Wavelet denoising [67] and the Weiner filters [68] can also be used for the ENG signal processing. A custom-made filter can be designed if the muscle activity potentials or the nerve tip information is available a priori [62,65] in order to extract the ENG peaks from the recorded noisy signal.

The ENG signal is the sum of many potential neuronal action potentials from a number of neurons, further signal processing is required in order to decompose the ENG signal into its contributing sources. The ENG signal is the sum of multiple action potentials, where each source signal has a different shape and time characteristic and thus it is possible to separate them. To obtain the individual sources of the composed ENG signal, several algorithms have been proposed in the literature. These algorithms are of the two basic types: one for identification of the peaks and second for their classification and decomposition. The signal is first preprocessed (band-pass filtered) to highlight the peaks, which detected and stored for later reconstruction. This method is known as alignment and is applied to detect and extract the identifiable peak properties. Once the peak is identified, the ENG signals are decomposed using a number of methods, such as template pairing or the convolution filtering [65], or wavelet transform [63]. The ENG signals often have the required low frequency (<50 Hz) modulation. Many signal processing methods have been used to extract the modulation signal, such as root mean square (RMS) signal or the absolute signal value through a moving window, Gaussian filtering [69], or Kaiser filtering [70]. The summary of the ENG signal processing methods is presented in Table 2.

## 4. Electrogastrography

**Figure 6:**
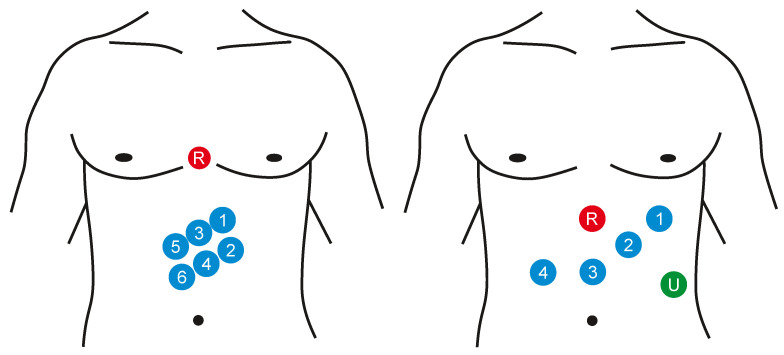
Examples of the surface placement of the EGG electrodes: signal electrodes (1–6), reference electrode (R), and ground electrode (U).

Electrogastrography (EGG) is a diagnostic method for recording bioelectric potentials of the stomach. The order of the measurement procedure is usually related with the examination of the motility (momentum, movements of the vegetative system realized by smooth muscles) of the gastrointestinal tract [6,71,72]. The EGG signal can be measured in vivo or on the surface (noninvasively) [72]. In case of the intragastric examination, the calomel electrode is induced into the stomach and the quality of the connection with a gastric mucous membrane is secured with the use of the saline solution. The reference electrode is connected to the upper limb. Because the proper positioning of the electrodes in the stomach is quite complicated and the presence of the foreign object affects its activity, it is possible to use only one electrode for the measurement procedure. When percutaneous recording, the electrodes are placed on the abdomen. It is not very difficult in the preparation, it is also faster and more gentle for a patient. It also enables using more channels for the recording and to observe better registration of the stomach motility (e.g., emptying). Either the monopolar or bipolar measurement can be applied, the reference electrode is placed on some of the hips, to armpits, or also on the abdomen. The standardized way of the EGG measurement has not been defined, so there is neither a unified system of the electrode localization nor the record length and ways to activate the gastrointestinal tract. Usually the 8 measuring channels are used in the above mentioned bipolar connection, but in general it is possible to use from 3 to 12 channels (see Figure 6). Some configurations use cutaneous reference points as landmarks for stomach shape and position while others take into account the actual position and shape of the stomach evaluated with the means of imaging diagnostics (X-ray or ultrasound) [6,7,71,73].

### 4.1. EGG Recording

**Figure 7:**
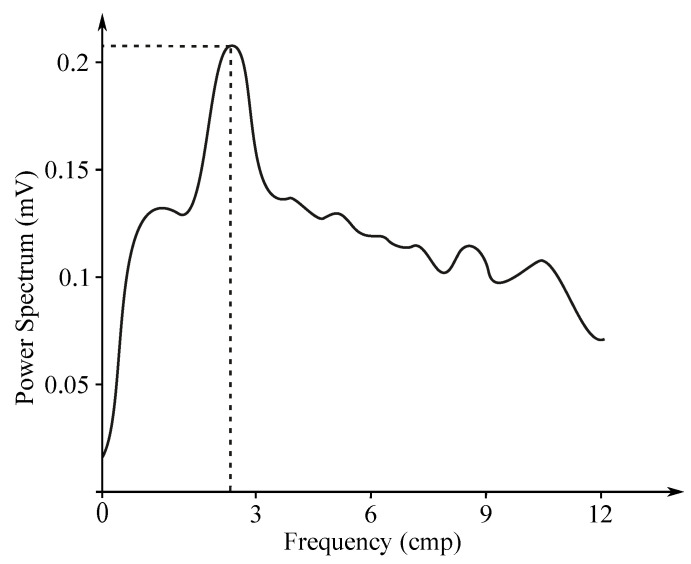
Sample EGG frequency spectrum.

Gastric electrical activity consists of spontaneous rhythmic electrical activity, which determines both timing and frequency ranges of the contractions. The dominant pacemaker of the stomach is placed along the greater curvature in the corpus and spreads towards the antrum and the pylorus. The pacemaker area is the dominating one due to the highest frequency of slow waves generated in the corpus. The slow wave frequency in the corpus pacemaker is approximately 3 cycles per minute (cpm). The overall frequency spectrum of the EGG signal is very low, within the range from 0.0228 to 0.15 Hz (0.5–9 cpm) (see Figure 7).

**Figure 8:**
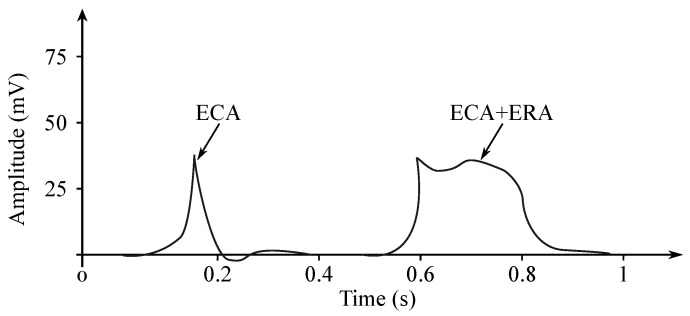
EGG potentials.

The EGG signal originates from the two types of the stomach electrical activity (see Figure 8): Electrical control activity (ECA) is the controlling activity and it begins in the gastric pacemaker, which is located on the low gastric wall and indicates the frequency of stomach contractions. The other type, the electrical response activity (ERA), is the result of the contraction of the smooth gastric muscles. It follows just after the ECA. The ECA occurs with a period of about 20 s and its repetition rate is very low, therefore it is given in cpm. In case of the repetition rate lower than 3 cpm, it is possible to assume a bradygastria, otherwise (the rate higher than 3 cpm)—a tachygastria.

The amplitude reaches values of about 80 mV in case of the in vitro measurement carried out in cells, while in case of the intragastric measurements it ranges from 0.1 to 10 mV and when recorded from the abdomen the amplitude range is only 10 to 500 µV. The interpretation of the EGG signal is very difficult, and the typical signal is not always legible from the background noise, making the manual analysis impossible. Therefore, the frequency analysis is usually applied as it provides more objective quantification [72]. A typical technique used for automated signal analysis is the running spectrum analysis, using the FFT applied in order to shorten the signal’s segments. The frequency analysis includes the following parameters: dominant frequency and power, fasting-fed power ratio, percentage of normal gastric slow waves, percentage of gastric dysrhythmias, and percentage of power distribution. Recording of the EGG signal is considered to be a supplementary examination method and is used at the specialized clinics for the purpose of studying the functional failures of the gastrointestinal tract [6,7,71,73,74,75,76].

### 4.2. Clinical Applications

Applications of EGG can be found in many areas of electrophysiological studies to assess the success of an intervention, effect of stress, or efficacy of pharmacological therapies, such as prokinetic therapies. The detection of EGG slow waves and their abnormalities allows evaluating the pathophysiology of gastric motility and contractile activities, whose increase correlates with the relative increase in EGG dominant power. Abnormalities in gastric motor function can indicate many disorders, such as functional dyspepsia, diabetes, diabetic neuropathy, systemic sclerosis, scleroderma, Parkinson’s disease, pseudo-obstruction, or experimentally induced motion sickness [77].

The EGG analysis is advantageous to combine with other techniques, such as manometry or gastric emptying measurement. For example, the postprandial change in the EGG dominant power has shown a significant correlation with the rate of stomach emptying in pediatric patients with gastroesophageal reflux. Similarly, dyspepsia can be assessed using gastric emptying scintigraphy. Furthermore, gastric dysrhythmias can also be detected from EGG, providing information on gastric hypomotility or impaired gastric contractions [78].

Many studies are based on the increased presence of abnormal frequencies in the EGG recordings. However, these abnormal frequencies are present also in healthy subjects, so the overlap between values recorded in diseased patients and healthy subjects must be taken into consideration [75,76,79].

### 4.3. EGG Signal Processing

In case of carrying out measurements from the surface of the patient’s body, the obtained signals also contain unwanted components obtained from the other organs or anatomy systems. These signals are the components of the EGG, but these can also be the following: baseline drift, poor contact of the sensor with the patient’s skin, or respiratory disturbances. Because the respiratory signals overlap with the EGG signal in the frequency range of 0.1 to 0.5 Hz, the breathing signal separation using the classical filtering methods leads to the distortion of the desired EGG and important information loss. Therefore, the EGG signals processing becomes quite complicated due to the presence of these artifacts and also the weak amplitude of the original signal [80].

Under laboratory conditions, the EGG studies are usually performed with subjects in a supine or stable sitting position, because of the extensive occurrence of motion artifacts. However, this position has the influence on autonomic gastric myoelectrical activity due to its non-physiological nature. Although small recording devices were developed in order to carry out measurements outside the laboratory settings, the movement-related artifacts remain a large and challenging problem, and their elimination is essential for the whole analysis procedure [75].

As the cutaneous EGG is a noninvasive technique, it is negatively affected with several limitations. Computer analysis of the EGG provides few reliable parameters, such as the frequency and the percentage of both normal and altered slow wave activity (bradygastria and tachygastria). New EGG hardware and software, along with the appropriate arrangement of the abdominal electrodes, could help to detect the coupling of the gastric slow wave from the EGG. Currently, the EGG does not allow diagnosis of a specific disease, but it puts in evidence the stomach motor dysfunctions in different pathological conditions as gastroparesis or functional dyspepsia [73,77].

A new approach for the EGG signals recording with high sampling frequency of 200 Hz was proposed in [71]. The high sampling frequency allows collection of signals, which include not only the desired EGG components, but also signals generated with other parts of the digestive system such as the duodenum and colon as well as signals emanating from the respiratory movements and ECG. The presented method in [71] allows for improvement of the quality of the analysis of the EGG signals with better suppressing respiratory disturbance and to extract new components from the highly sampled EGG signals obtained from the surface of the abdomen. The source of the required new signal’s components can be inner organs such as the duodenum and colon, which were mentioned above.

#### 4.3.1. Adaptive Filtering

The signal’s quality improvement with the implementation of the adaptive filters was proposed back in 1990 [81], when the SNR of the EGG signal was increased and some artifacts were eliminated, including the respiratory artifact, ECG, and noise resulting from electrode–skin interface and some motion artifacts. Another investigation of the LMS adaptive filtering focused on the respiratory artifact elimination [80], when the reference respiratory signal was recorded using a thermistor sensor. The method presented in [80] showed promising perspective for the suppression of respiratory components. However, the performance of this adaptive method depends on the selection of the appropriate filter parameters, which could be the subject of further interesting investigations.

#### 4.3.2. Wavelet Transform

Qiao et al. [82] presented an application of the continuous WT in the EGG signal analysis, which gives a better time-frequency resolution than the short-time FT in non-stationary signals, such as the EGG. The WT method provides good frequency resolution and enables the monitoring of the evolution of the slow wave and the other EGG components. Furthermore, the continuous WT enables locating high frequency variations or spike activity.

Ryu et al. [83] evaluated the performance of the WT filters in comparison with the use of the classical FIR and IIR filters. The authors tested different filter coefficients considering their efficiency, performance and filtering pace using the Daubechies wavelet. Based on the SNR and the reconstruction squared error calculation, the WT filters performed better than the other various types of the DFs.

The problem of finding a wavelet, which best “matches” the wave-shape of the EGG signals in basal state were presented in [84] with preprocessing the signal within the range of 0.02 to 0.2 Hz—the low-pass first-order Butterworth active filter. This study focused on the selection of appropriate parameters, such as number of scales, compression ratio, and optimization of the wavelet choice. The proposed optimal wavelet showed similar performance to the well-known and widely used Daubechies-3 wavelet, which can be chosen instead of the above proposed wavelets.

#### 4.3.3. Independent Component Analysis

Wang et al. [85] proposed the EGG signal separation from the combined signal measured from the abdomen using the ICA. Three independent components were extracted: the EGG signal, respiratory motion, and other interference from the environment. As ICA does not require the reference signal (contrary to the ANC), it seems to be easier to implement and it converges faster.

Hubka et al. [86] proved the usefulness of the ICA separation of the EGG signal from the mixture of the desired slow wave and the background noise. They used four independent components in order to extract the EGG signals correlating with the first component.

#### 4.3.4. Empirical Mode Decomposition

Liang et al. [87] extracted the EGG signal from the corrupted recordings with the implementation of the EMD method and the Hilbert transform. Based on the frequency content of each extracted component, the first three extracted functions corresponded with heartbeat, respiratory artifact and harmonic signal, and the fourth function was assumed to be the gastric slow wave. Because the measurements were performed using a portable EGG recorder, motion artifacts occurred in this approach, which was very challenging. The authors of that solution also presented the difference in artifacts suppression using the EMD and the classical DF methods, where the spectrum of the EMD signal extracted was significantly less distorted in comparison with the signals filtered using the band-pass DF.

#### 4.3.5. Hybrid Methods

In the analysis of biomedical data, it is very challenging to remove undesirable components while leaving those necessary for analysis purposes [33]. Therefore, the implementation of the appropriate filtering methods is in the highest demand. The authors of this work have comprehensive experience with biomedical data, which was related to inter alia implementation of smoothing filters or non-integer order filters [14,88]. Popovic et al. [89] combined a smoothing filter (Savitzky–Golay) with non-integer order filtering for the purpose of analysis of the EGG data. No similar approach has been found in the literature and the obtained results were very interesting.

Komorowski et al. [74] proposed a new approach for denoising of the multichannel EGG signals with the implementation of the Noise-Assisted Empirical Mode Decomposition (NA-MEMD) and adaptive filtering. The method is based on extracting the reference signal for the adaptive filter by NA-MEMD and tested on real human EGG signals and EGG signals with added synthetic noise. The obtained results were compared with classical band-pass filtering, ICA, and adaptive filtering. The proposed method proved its efficiency by promising values of SNR and the basic diagnostic parameters used in EGG analysis, i.e., the dominant frequency, the normal gastric rhythm index, the frequency instability coefficient, and the power instability coefficient.

Sengottuvel et al. [90] combined ICA and EEMD signal separation methods to reach a better elimination of cardiac, breathing, and drift artifacts and lower signal distortion compared to conventional techniques, such as band-pass filtering, ANC, and EMD-based methods. The evaluation of the performance of this hybrid method was performed using reference sensors measuring body movements of the abdomen (accelerometric sensor) and respiratory activity (thermistor sensor). The correlation of these signals with the residual signals (obtained by subtracting denoised signals from the raw EGG data) was then investigated for each method.

Table 3 presents a summary of the most common EGG signal processing methods.

## 5. Electrooculography

**Figure 9:**
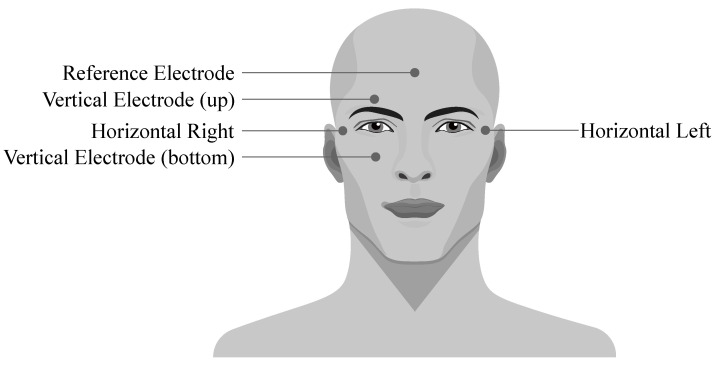
Placement of the EOG electrodes.

Electrooculography (EOG) is a diagnostic method of recording the electrical activity of eyes. It is based on the fact that an eye cornea has a positive charge and retina has a negative charge (so the eye bulb represents an electrical dipole). This enables the genesis of the potentials which cause changes in electrostatic field when the eye changes its position. The dipole is orientated in accordance with the front-end axis of the eye bulb and its direction minimal deviates from the optic eye axis. During the movements of the eye bulb into sides, the size of the charge changes according to the size of the rotation. The EOG signal is recorded using the electrodes placed around the eyes which can be connected either in bipolar or unipolar positions (see Figure 9) [91].

Thanks to the EOG signal, it is possible to evaluate the function of eye muscles or protrusion of the eye from the orbit; monitor the eye movements during sleep, anesthesia, or diagnosis of some vascular disorders; and retinopathy. The analysis of the lateral eye movements is at most frequently applied in psychophysiological methods such as speech therapy, evaluation of the stress, emotions, fatigue, or treatment with psychopharmaceuticals [7,92].

### 5.1. EOG Recording

**Figure 10:**
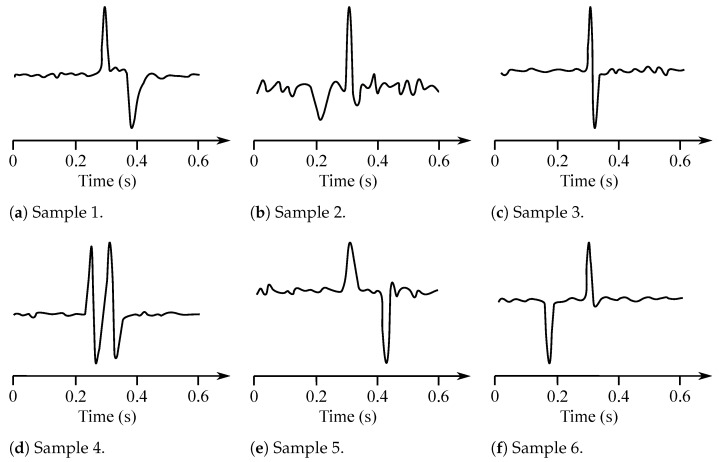
Sample EOG sisgnals.

**Figure 11:**
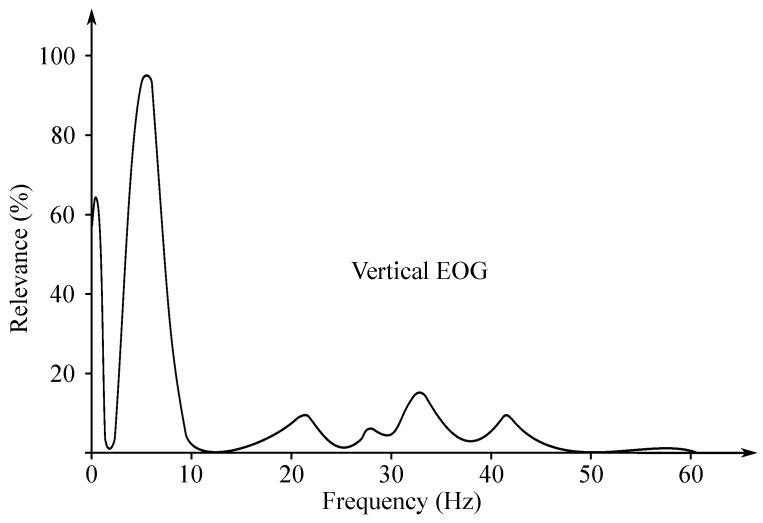
Sample EOG spectrum.

The EOG signal belongs to the group of random (non-stationary) signals, as their spectrum vary over time [93]. Its frequency ranges from 0.5 to 15 Hz and is characterized with a significant DC component. Values of the amplitude do not exceed mV range, but usually are lower than 2 mV (around 50–3500 µV). The change of the voltage is caused by the 1-degree change in view angle [7]. Samples of the EOG signals are illustrated with Figure 10, while Figure 11 presented the EOG spectrum.

### 5.2. Clinical Applications

In some cases, the EOG is a useful tool applied for the diagnostics purposes of inherited macular diseases [94]. Understanding the disease states that the affected with the disease EOG helps with the interpretation of the results. In conjunction with the ERG, it may be useful in the process of diagnosis of various progressive retinal disorders.

Typically, the EOG is a method used for the purpose of eye movements recording during the ENG, except for the classical EOG, which uses direct current amplification (direct current), while the ENG in clinical practice often uses alternating current amplification (alternating current capacitor) with a time constant of 5 or 10 seconds, which results in a high-frequency filtered signal where slow fundamental shifts are damped.

One of the signals that can be detected using the EOG method is the eyeblink. In some cases, this signal is considered as the desired one, often used as means of communication with paralyzed individuals [95,96], whereas in others as an artifact and thus should be removed.

Finally, among the most recently discussed applications of EOG is a so-called ’eye-writing’, where the EOG-based method can be used as an alternative to conventional camera-based eye trackers. The feasibility of the EOG-based eye-writing as a new communication approach for individuals with amyotrophic lateral sclerosis (ALS) was investigated in [97,98]. In [97], the authors developed an EOG-based eye-writing system and tested it on 21 participants (18 healthy and three with ALS). The system achieved a mean recognition rate of 95.93% for healthy participants and 85% for subjects with ALS. In [98], the authors evaluated the eye-writing system with a symbol set consisting of symbols of 10 Arabic numerals and four mathematical operators. Experiments on 11 voluntary human subjects showed recognition rate ranging from 50% to 100% with different symbols.

In [99], the authors proposed a continuous eye-writing recognition system that receives eye-written characters continuously and thus achieves a high input rate. This system detects eye movements by EOG and then applies a hidden Markov model (HMM) to model the EOG signals and recognize the eye-written characters. Experiments with six participants showed an average input speed of 27.9 character/min.

### 5.3. EOG Processing Methods

In the EOG signal, the most common artifacts are (except the PLI or other noise coming from the electromagnetic fields) baseline drift, neck movement (resulting in the low-frequency changes in the signal), muscles potentials around the eyes (having the maximum power in the frequencies above 20 Hz), eye blinking (random potential differences in frequency range of 0.5 to 3 Hz), and saccades. Moreover, the simultaneous activity may cause the electrodes to lose contact or move on the skin surface, which decreases the data quality in a significant way [100,101].

The EOG signals can be efficiently applied in various Human–Machine and/or Human–Computer Interfaces, as they are usually based on users’ eye movement intentions or actual movements, thus such systems use limited number of commands based on basic eye movements (looking up, down, left, or right) [102,103,104,105].

He et al. [105] proposed a novel single-channel EOG-based solution, which allows users to spell with only blinking. In his system, forty buttons correspond with the 40 characters displayed to the user in a random order, where the user must blink when the target button flashes. The authors used two different signal processing methods—the support vector machine (SVM) classification and the waveform detection, which were combined for the purpose of the eye blinks detection. The obtained experimental results demonstrated the effectiveness of the proposed solution with an average accuracy of 94.4% and a response time of 4.14 s.

Typical signal processing algorithms frequently suffer from large execution delays, which is challenging or makes it even impossible for the real-time analysis—therefore, implementation of high-speed algorithms is needed. Agarwal et al. [92] implemented a multiplier Savitzky–Golay smoothing filter (SGSF) based on distributed arithmetic (DA) for the purpose of the EOG signals preprocessing, so that the processing speed has been increased along with the necessity for the chip area reduction. The filter applied for this purpose should be efficient enough in order to remove the artifacts along with least deformation or affecting of the actual signal in a negative way. The Savitzky–Golay (SG) filter is widely applied in analysis of biomedical signals [88]; however, despite it being fast and efficient and easily implementable, it has not been applied for the purpose of the EOG analysis so far. The SGSF is sometimes chosen for the purpose of accurate diagnosis using saccade detection of the EOG signals. The efficiency of the proposed filter was tested in terms of the signal-to-signal-plus-noise ratio (SSNR) and the real-time computations. It was observed based on the performed analysis that the DA based architecture increased the processing speed, reduced the chip area and the original features of the filtered signal were preserved. In Table 4, a summary of EOG signal processing method was presented.

As for the detection or extraction of the eye blink, various methods have been used in the literature, such as WT [106,107], evaluation with thresholds [108,109], derivation of the signals [110,111], or analysis of EOG velocity based on expert rules [112]. Moreover, additional features of the blinks, such as start time, speed, or duration of the eye closure, can be obtained using advanced signal processing and classification methods [113,114,115]. In contrast, there are areas where the eye blink must be eliminated, mostly from the EEG signal [116,117,118,119]. For example, in [120], the authors introduced a novel method for EEG filtering based on signal modeling, time variant covariance matrices, and Kalman filter. The eye blink model was created using a single channel EOG.

## 6. Electroretinography

**Figure 12:**
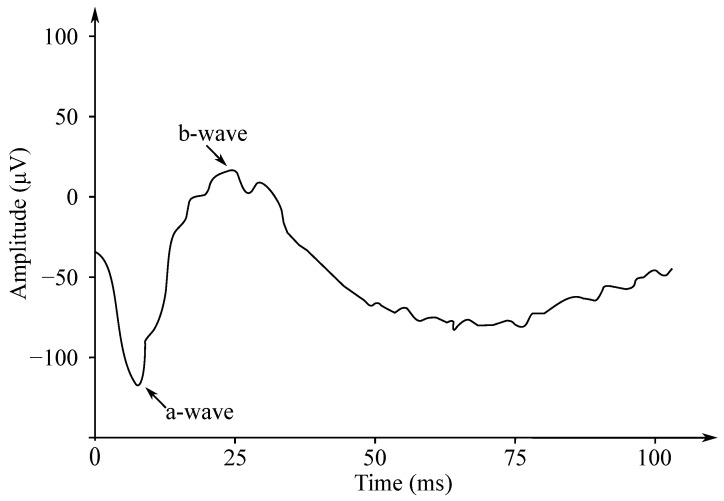
The biphasic waveform of a healthy patient—the negative wave (**a**) and the positive wave (**b**).

Gotch in 1903 [121] was the first to state that the eye’s response to a flash of light consisted of the two waves; the first one was negative, while the second one—positive (with a greater amplitude). Later, Einthoven et al. in 1908 [122] divided the ERG response into the three waves: The first wave, which appeared immediately after turning on the light stimulus, was negative on the cornea and was followed with a positive wave. The last wave was slower, but also positive. Einthoven et al. suggested that the light stimulus triggered a chain of reactions leading to the formation of the products A, B, and C, and that each electric wave indicated a change in the “relevant” product. The work of these authors was the basis for starting the research on the ERG signals’ processing, analysis and implementation, and these are used to this day. The waves are called a-, b- and c-waves. Another positive cornea wave, which is less frequently recorded at the end of a flash of light, is called the d-wave. In Figure 12 the biphasic waveform of a typical healthy patient was presented.

The electroretinography (ERG) is a diagnostic test, which enables measurement of the electrical activity of the retina in response to a light stimulus. It is based on currents generated directly by retinal neurons in combination with the contributions from the retinal glia. The ERG is an objective measure of the retinal function, which can be recorded noninvasively under physiological conditions. The ERGs are often recorded using a thin fiber electrode which is placed in contact with the cornea or an electrode which is built into the contact lens of the cornea. These electrodes enable recording of the electrical activity generated by the retina on the surface of the cornea. The ERG can be induced with diffuse flashes or patterned stimuli [123].

### 6.1. ERG Recording

For the ERG recording purposes various locations of the recording electrodes placement: in contact with cornea, bulbar conjunctiva, or skin below lower eyelid. It is possible to distinguish the following types of the recording electrodes [123,124]:

- Burian–Allen (BA): made of a stainless-steel annular ring surrounding the core of the polymethyl methacrylate (PMMA) contact lens. The BA electrodes contain a lid mirror in order to help to minimize blinking. They are reusable.
- Dawson–Trick–Litzkow (DTL): low-weight electrodes made of conductive silver or nylon fiber. They are disposable and are usually more convenient for patients.
- Jet: disposable plastic lens with gilded peripheral circumference.
- Skin electrode: can be used as a replacement for corneal electrodes by placing the electrode on the skin over an infrared comb near the lower lid. The ERG amplitudes tend to be small and prone to noise occurrence, but they are better tolerated in pediatric populations.
- Mylar electrode: Allarized or gold-plated Mylar, not commonly used.
- Cotton-wick: A Burian–Allen electrode sheath equipped with a cotton wick, which is useful for minimizing light-induced artifacts, not commonly used.
- Hawlin–End Electrode: Teflon insulated thin metal wire (silver, gold or platinum) with three central windows, length 3 mm, shaped to fit in the lower conjunctival sac, are not commonly used.

### 6.2. Clinical Applications

The ERG signals are mainly used by ophthalmologists and optometrists, and are used for the diagnostics purposes of various retina diseases. The ERG has important clinical applicability in providing diagnostic information for a number of inherited and acquired retinal disorders. In addition, the ERG can be used for monitoring disease progression and evaluation of the retinal toxicity due to usage of various drugs or presence of foreign bodies.

Other ERG assays, such as the photopic negative response (PhNR) and the ERG model (PERG), may be useful in assessing retinal ganglion cell function in diseases such as glaucoma. The multifocal ERG is used for recording of separate responses for different retinal sites.

### 6.3. ERG Signal Processing

The ERG signals have two important amplitudes used for various disease diagnostics by medical professionals. These are the negative a wave and the positive b wave. The implicit wave times a and b are also useful for diagnostics purposes. The ERG signals have small amplitudes (about µV). For this reason, it is important to separate the signal from the noise and from the interference, which may occur due to movement.

An artifact may appear in the ERG, which may interfere with the recording and interpretation of the ERG b wave. This artifact is a photomyoclonic reflex (PMR) and was studied by Johnson et al. [125], who during their experiments covered the eye containing the recording electrode and stimulated the other eye at the same time. The recordings obtained with the implementation of this technique before and after administration of the modified Van Lint eyelid block showed that most PMRs are caused by reflex contraction of the orbicularis muscle. The remainder of the PMR was detected through recording the eye movement 1.5 to 3.5 degrees down and with the observation of the medial eye movement.

The line noise refers to the electrical interference induced in the cable connecting the electrode with the amplifier. Because the input impedance of the amplifier is high, noise can be generated in the cable with the capacitive or magnetic couplings coming from the surroundings. These effects can be limited to some extent with the usage of shielded or twisted cables. The main type of the noise interference comes from 50 or 60 Hz generated with the power lines and electrical outlets. This lies within the bandwidth ranges (1–300 Hz) of the electromagnetic signal (the ERG), and therefore severely impairs the quality of the recorded data. Many electrodiagnostic recording systems use active electrodes in order to overcome the line noise. This involves connecting the first amplifier as close as possible to the recording electrode. The electrical noise can be reduced with the impedance transformation because the low output impedance of the amplifier is almost impermeable to the electrical or magnetic interference [126].

Latifoglu et al. [127] proposed to use empirical mode decomposition in order to denoise the ERG responses. The ERG signals are the non-stationary signals that are decomposed into a number of intrinsic mode functions, so then the noise and interference can be eliminated. Finally, the ERG signals having their signal-to-noise ratio of less than or equal to 10 dB are reconstructed, which enables them to obtain the denoised ERG signals [128].

Santiago et al. [129] used a method for processing the multifocal electroretinogram (mfERG) recordings in order to improve the ability for diagnosing the MS. They examined the mfERG recordings obtained from 15 patients with early-stage MS without a history of optic neuritis and from 6 healthy participants (control subjects). The mfERG recordings were filtered using the EMD. Correlation with the signals in the normative database was used as a classification function.

The Discrete Wavelet Transform (DWT) is a fast and efficient analysis method for the ERG, which reveals time and frequency information regarding the examined signal. The choice of parent ripple is important for the best extraction of the desired components. In [130], optimization of the selection of the Daubechie Wavelet for the ERG collected with the Photopic Negative Response (PhNR) stimulus through variable evaluation and selection of functions in the classification of glaucomatous and non-glaucomatous eyes is presented.

Visual function testing using the ERG signals is used in order to detect retinal abnormalities. This is achieved by measuring, characterizing and analyzing biopotential responses from various retinal cells generated by visual stimulation. The aim of this study was to improve the already existing models of the ERG signal characteristics with the identification and inclusion of the key components called i-waves in the photopic response. In order to verify the proposed characteristics model—Adithya et al. [131] developed a signal analysis and processing algorithm based on the multi-resolution analysis, which reliably separated various basic components of these signals. Finally, the accuracy of this separation was assessed quantitatively and qualitatively by calculating the Pearson correlation coefficient and the corresponding scatter plots between the composite and the reconstructed ERG signal.

Retinitis Pigmentosa (RP) is one of the degenerative diseases of the retina affecting the eye signals. The ERG is a signal, which plays an important role in the diagnosis and treatment of the RP. This signal contains useful information, which cannot be detected only in the time domain. Ebdali et al. [132] investigated the influence of the RP on the time, frequency and time-frequency parameters of the ERG using the Fourier and wavelet transform methods. In Table 5, a summary of signal processing methods of the ERG data is presented.

## 7. Electrohysterography

Monitoring of the uterine contractions is commonly used in order to determine whether childbirth is coming. In the beginning, the uterine activity is weak and localized, but with increasing pregnancy duration, the contracting gradually becomes stronger and stronger, rhythmical, and well propagated. Nowadays, the intrauterine pressure catheter (IUCP) is a golden standard for the monitoring of the uterine contractions, but it requires membrane rupturing, so it can be used only during labor and it carries a risk of the intrapartum infection. For the noninvasive monitoring of the uterine contractions, a tocodynamometry is commonly used in clinical practice in order to determine both frequency and duration of the contractions. Unfortunately, this approach is inaccurate, uncomfortable, and is particularly dependent on the subjective evaluator’s (medical professional) assessment [133,134].

Electrohysterography (EHG) is a noninvasive method of sensing the electrical activity of the uterine contractions recorded from the electrodes placed on the maternal abdomen. This method has been known for more than sixty years and provides valuable information for evaluation of the contraction intensity and strength. Note that the uterine electrical activity changes during pregnancy and when the birth is coming. This is reflected in the temporal and spectral characteristics of the EHG signals. Some papers discuss that both the velocity and the direction are associated with the contraction efficiency. However, the signal resulting from the EHG contains a lot of artifacts, so it is difficult to estimate the useful information. Moreover, there is still no standardized approach to the EHG signal processing and acquisition [135,136,137,138].

### 7.1. EHG Waveform

**Figure 13:**
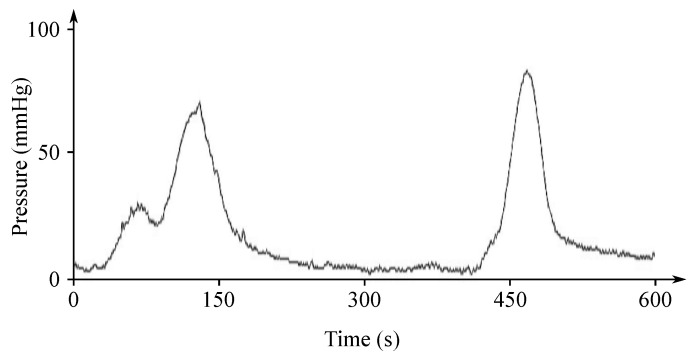
Plot of a recorded IUCP signal.

**Figure 14:**
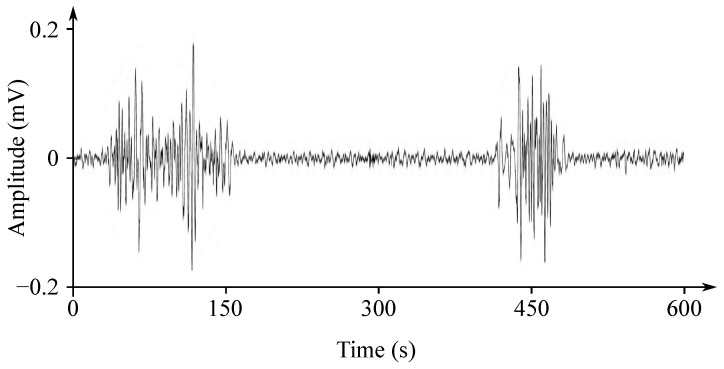
Plot of a recorded EHG signal.

**Figure 15:**
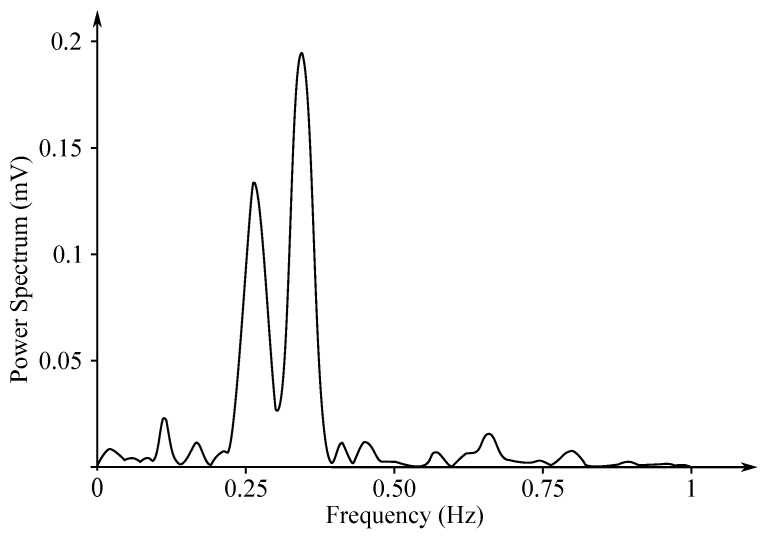
Power spectrum of the first contraction from the Figure 14.

The EHG signal is recorded with the implementation of the abdominal electrodes and could be described as a slow electrical wave with the frequency within the ranges of 0.03 to 0.1 Hz and amplitude of 1–5 mV. The fast-electrical activity is superimposed on this slow electrical wave with the frequency of 0.3–2 Hz and the amplitude 50 µV–1 mV. In the spectral domain, the EHG signal lies in the interval between 0.1 and 3–5 Hz [139,140]. Figure 13 and Figure 14 show plots of the IUCP and the EHG recorded signals and Figure 15 shows the power spectrum of the first contraction from the Figure 14.

**Figure 16:**
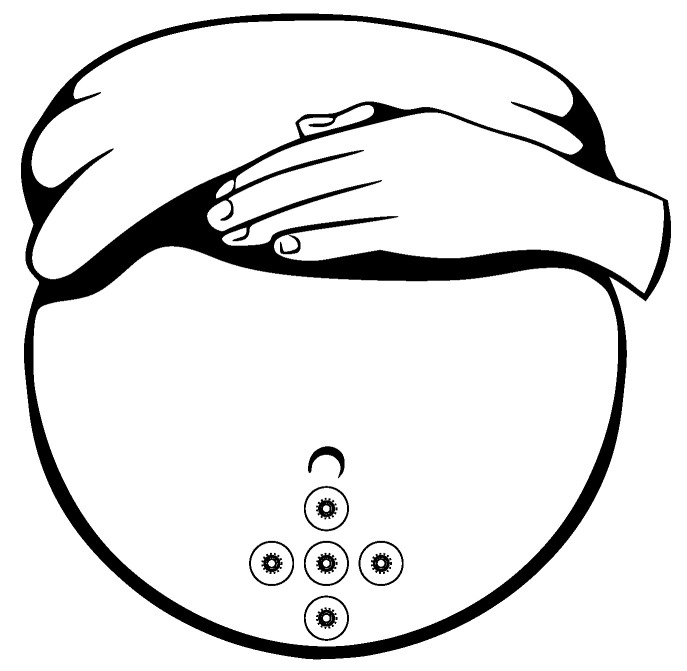
Configuration of location of the electrodes for the EHG acquisition.

Gondry et al. in 1993 [141] showed that the measurement of the EHG signals could be performed as early as at the 19 weeks of pregnancy. For the noninvasive EHG measurements there are commonly used Ag/AgCl electrodes (8 mm diameter). An example of the appropriate electrode placement for the EHG is shown in Figure 16 [139,142]. Configuration and placement of the electrode in the region immediately below the umbilicus provides the best SNR. For the EHG measurement purposes the 25 mm interelectrode distance is commonly used, the reference electrode is placed on the right hip and the ground electrode is placed on the left hip [139,140,143,144,145].

### 7.2. Clinical Applications

Currently, this promising noninvasive method of sensing the uterine contractions is not used in clinical practice due to the difficulties involved in interpretation of the information contained in the EHG signal. This signal contains a lot of interferences coming from both mother and fetus. Various extraction techniques were used in order to improve the quality of the EHG signals and the automatic detection of the birth or pregnancy related contractions. Some studies [146,147,148] show that the EHG seems to be a more accurate alternative method for the IUCP than the tocodynamometry, especially for women with a higher BMI. However, although the EHG accurately detects the uterine contractions and the complexity of the contraction’s characteristics obtained with the EHG, which cannot be compared to the IUCP [149].

Parameters of the EHG signals obtained during contractions could be used for the purpose of diagnosis of the preterm labor coming and to predict preterm delivery. Great attention has been recently paid on predicting labor and discriminating preterm contractions based on the information received from the EHG signals. Some preterm contraction will lead to the preterm delivery, but some others will not, which makes their appropriate interpretation a very challenging task. The parameters derived from the EHG signals and related with their frequency are expected to be more comparable from one subject to another than the parameters related with the amplitude and less sensitive to the electrode placement [133].

### 7.3. EHG Signal Processing

The EHG is a very problematic signal because it has a very low frequency and a very low amplitude often leading to its confusion with different noise and interferences. This signal contains the uterine electrical activity (contractions) but also a lot of interference, such as those caused by the abdominal muscle activity, the baseline fluctuations, or the motion artifacts [139,148,150]. Presence of these artifacts could lead to distortion of the signal’s spectral power density and to the unreliable (inefficient) automatic identification of the contractions obtained from the EHG signal. The motion artifacts are the main problem in the analysis of the EHG signals. However, also a great attention is needed to to be put on the fECG and the mECG signals. It needs to be realized and taken into account that the noise present in the monopolar EHG is non-stationary and has usually much higher amplitude than the signal of interest. In addition, the above-mentioned interferences have their main frequency component very close to the frequency of the EHG signal.

The interference signals are usually overlapping the EHG signal of interest in both time and frequency domain, therefore it is necessary to reduce or completely eliminate them. Nowadays, various advanced signal processing methods are used in order to extract the EHG signal from signals measured with the electrodes placed on the maternal abdomen. Very often the various digital filters—EMD, ICA, and WT—are used for the purpose of interference components elimination from the measured signals and in order to estimate the appropriate EHG signal. Very promising results are given by some modern hybrid methods, especially based on the combination of the EMD with some other methods. Table 6 shows a summary of methods for the EHG signal processing.

#### 7.3.1. Linear Filtering

Ye-Lin et al. in 2014 [139] used a 5th-order Butterworth bandpass digital filter with its cut-off frequencies at 0.1 and 4 Hz for the purpose of the data denoising. They also downsampled the analyzed signals to only 20 Hz in order to decrease the computational cost. They concluded that such a low sampling frequency is sufficient for the computation of the spectral parameters and that the applied Butterworth bandpass digital filter is suitable for the purpose of the data preprocessing. Garcia-Gonzalez et al. in 2013 [151] used a bandpass digital filter with its cut-off frequencies at 0.3 and 4 Hz to denoise the EHG signals, where Acharya et al. in 2017 [152] used a 4-pole bandpass Butterworth filter with the cut-off frequencies at 0.3 and 3 Hz to carry out the preprocessing of the EHG signals.

#### 7.3.2. Wavelet Transform

Leman and Marque, in 2000 [153], tried to eliminate the ECG signal from the measured EHG signals on the maternal abdomen with the implementation of the WT. For their experiments they applied simultaneously recorded mECG signals with the EHG signals. Their proposed algorithm was based on the local minima detection in the histogram. They concluded that the ECG signal was very efficiently removed from the EHG data and improved its overall quality and eligibility for further processing.

Beiranvand et al., in 2017 [154], used the DWT in order to extract some features from the EHG signals. After performing the DWT, the supporting vector machine technique was used in order to classify the analyzed signals. Real data from an open access database, The Term-Preterm EHG, was used in their study, where they chose 26 recordings from the term delivery and 26 recordings from the preterm delivery. For the evaluation purposes the calculation of relative wavelet energy, root mean square, accuracy, sensitivity, and specificity were applied. They came to the results that the best accuracy was achieved with the use of the 4th level DWT decomposition and with the maternal wavelet db2.

#### 7.3.3. Empirical Mode Decomposition

Taralunga et al., in 2015 [155], proposed the EMD method for denoising the EHG signals. The main advantage of their method is that no a priori knowledge regarding the signal is required, because it is fully data-driven. For the experiment purposes, the synthetic data and the calculation of the SNR improvement were used. They concluded that the EHG signal has a significant predictive value in case of a preterm labor. The authors also mentioned that when the SNR of the input signals is low, then the EMD method could be combined with another method in order to improve the overall performance.

Ren et al. in 2015 [156] used the EMD method for the risk of preterm delivery assessment based on the information obtained from the EHG signals. The real data used in their studies was acquired from the open access of the Term-Preterm EHG database (262 term and 38 preterm). After performing the EMD for the purpose of the extraction of the IMF, the instantaneous amplitude and the frequency of each IMF component were computed. Area under the curve (AUC) values was used as an evaluation parameter in that study. They came to the conclusion that their approach improves prediction accuracy of the preterm delivery risk compared with some previous approaches based on the high value of the AUC.

#### 7.3.4. Hybrid Methods

Chkeir et al., in 2010 [157], proposed the EHG enhancement method based on the combination of the EMD-based method with the SG filter, which enabled to remove the high-frequency noises and the baseline wander with a minimal signal distortion. They used the real data provided by the Landspitali University hospital in Iceland (they selected 10,000 random contractions) and various artificially generated signals in order to evaluate the performance of the proposed method. For the purpose of evaluation, a SNR improvement calculation was performed. The authors concluded that their approach provided good results compared with the WT method, with the advantage of being adaptive and with no need for pre-definition of the parameters.

Hassan et al., in 2011 [158], used a combination of the canonical correlation analysis (CCA) and the EMD to denoise the monopolar EHG. The CCA method belongs to the group of the BSS methods. Their method was compared with the other BSS methods (ICA, PCA, etc.), while their approach solves the main BSS problem by forcing the sources to be maximally autocorrelated and mutually uncorrelated, while the mixing matrix is assumed to be square. First, they used the CCA in order to extract the uterine bursts, and then the EMD was used for removal of the biggest part of any residual noise from bursts. Their approach was compared with the ICA and with the WT. For the study purposes, the real data from the Landspitali University hospital in Iceland were used and the method’s accuracy was evaluated with the SNR improvement calculations. They concluded that the proposed method successfully removed artifacts from the signal without altering the underlying uterine activity. Other methods analyzed in that paper did not achieve comparable accuracy to the one obtained with the implementation of the proposed approach.

Acharya et al., in 2017 [152], proposed the combination of the EMD and the WT for the estimation of the premature delivery based on the information obtained from the EHG signals. Real data from the open access Term-Preterm EHG database were used in that study (262 term and 38 preterm). Calculation of accuracy, sensitivity, and specificity was used for the evaluation purposes. They concluded that the proposed method reached a very good accuracy of 96.25% and could be used in hospital gynecology departments in order to predict the preterm or normal delivery. Similar approach was used by Hoseinzadeh and Amirani in 2018 [159]. They also used the same database and evaluation parameters and concluded that this approach achieved a very high accuracy.

## 8. Discussion and Conclusions

This paper is a Part III work and focuses on a review of signal processing methods for other (remaining) bioelectrical signals, which do not belong to the group of signals related with heart or brain electrical activity.

The choice of the most optimal processing methods was based on the signal type, and the signals described in this work varied, which means they had among the other different frequency ranges, amplitude spectrum, etc.

As it was mentioned in other parts of this paper, typical, classical digital filtering methods can be applied when both the interference and desired biological signals have various frequency ranges. Therefore, using adaptive noise canceling techniques can be a good alternative to the traditional filtering methods.

The second group of the advanced signal processing methods does not require the reference signals and seems to be more sensitive to preserving the analyzed signal’s information both in the time and the frequency domain. One of the methods providing good time-frequency distinguish-ability is the WT. However, the optimal selection of the wavelet decomposition parameters and properties of the used maternal wavelet is crucial. In many studies, these properties are chosen empirically, because of various time and frequency characteristics of the signals recorded from different subjects. The choice of the appropriate WT parameters depends also on the type of biological signal. Therefore, the introduction of this method into medical practice is not yet well established, especially as the other methods such as ICA and PCA have provided better performance in eliminating artifacts overlapping the frequency spectrum. Their disadvantage is the necessity for the multichannel recordings, as the algorithms work with matrices of the signals. The ICA method has the advantage of enabling separation of the higher number of components than the number of channels (compared to the PCA method, where the number of channels gives the maximum number of components). On the other hand, the independent components are of different amplitude compared with the extracted signal, and their order is random for each calculation. Signal decomposition using the EMD method seems to be useful for the elimination of the narrow-band interference, especially the low-frequency components. However, the computational costs are quite high, which does not allow practical application of this method in the near future.

For what we have referred to as improved or combination methods, also known as hybrid approaches, the highest performance is in the elimination of the greater range of the interferences and artifacts. However, the computational costs and complexity of their implementation have caused these methods to currently remain in the research field. Their implementation in the biomedical devices for clinical implementations may come in the future. In general, the suitability of signal processing methods for their introduction into clinical practice depends not only on their performance, but more importantly, on their computational requirements, with the most critical one being the real-time implementation followed by the cost. For the feasibility of implementation into clinical practice, one should find a compromise between computational cost and performance. Presently, most of the advanced methods discussed in this paper are not suitable for clinical applications, mainly due to implementation challenges or financial cost of application. Still, it can be expected that the price of these digital techniques will become acceptable with the increasing development of microprocessor techniques over the next few years.

## Figures and Tables

**Table 1:** Summary of the EMG signal processing methods.

Method	Overall Performance	SNR Improvement	Computational Cost	Real-Time	Implementation Complexity
DF	Low	Low	Low	Yes	Simple
ANC	High	Medium	Medium	Yes	Medium
WT	Low	Medium	Medium	Yes	Medium
ICA	High	High	Medium	No	Medium
EMD	High	Medium	High	No	Medium
Hybrid Methods	High	High	Medium	No	Complex

**Table 2:** Summary of the ENG signal processing methods.

Method	Overall Performance	SNR Improvement	Computational Cost	Real-Time	Implementation Complexity
DF	Low	Low	Low	Yes	Simple
ANC	High	Medium	Medium	Yes	Medium
WT	Low	Medium	Medium	Yes	Medium
ICA	High	High	Medium	No	Medium
EMD	High	Medium	High	No	Medium

**Table 3:** Summary of the EGG signal processing methods.

Method	Overall Performance	SNR Improvement	Computational Cost	Real-Time	Implementation Complexity
DF	Low	Low	Low	Yes	Simple
ANC	Medium	Medium	Medium	Yes	Medium
WT	Medium	Medium	Medium	Yes	Medium
ICA	High	Medium	Medium	No	Medium
EMD	High	Medium	High	No	Medium
Hybrid Methods	High	High	High	No	Complex

**Table 4:** Summary of the EOG signal processing methods.

Method	Overall Performance	SNR Improvement	Computational Cost	Real-Time	Implementation Complexity
SVM	Medium	Medium	Low	Yes	Medium
SGFM	High	High	Medium	Yes	Medium
DA	Low	Medium	Low	Yes	Low

**Table 5:** Summary of the ERG signal processing methods.

Method	Overall Performance	SNR Improvement	Computational Cost	Real-Time	Implementation Complexity
DF	Medium	Low	Low	Yes	Simple
ANC	High	Medium	Medium	Yes	Medium
WT	Medium	Medium	High	Yes	Medium
EMD	High	Medium	High	No	Medium

**Table 6:** Summary of the EHG signal processing methods.

Method	Overall Performance	SNR Improvement	Computational Cost	Real-Time	Implementation Complexity
DF	Low	Medium	Low	Yes	Simple
DWT	Low	High	Medium	Yes	Medium
EMD	High	Medium	High	No	Medium
Hybrid methods	High	High	High	No	Complex
